# Looking for combination of benznidazole and *Trypanosoma cruzi-*triosephosphate isomerase inhibitors for Chagas disease treatment

**DOI:** 10.1590/0074-02760170267

**Published:** 2018-03

**Authors:** Elena Aguilera, Javier Varela, Elva Serna, Susana Torres, Gloria Yaluff, Ninfa Vera de Bilbao, Hugo Cerecetto, Guzmán Alvarez, Mercedes González

**Affiliations:** 1 Universidad de la República, Facultad de Ciencias, Grupo de Química Medicinal, Montevideo, Uruguay; 2 Universidad Nacional de Asunción, Instituto de Investigaciones en Ciencias de la Salud, Departamento de Medicina Tropical, Asunción, Paraguay; 3 Universidad de la República, Facultad de Ciencias, Centro de Investigaciones Nucleares, Área de Radiofarmacia, Montevideo, Uruguay; 4 Universidad de la República, Centro Universitario Regional Litoral Norte, Laboratorio de Moléculas Bioactivas, Paysandú, Uruguay

**Keywords:** Chagas disease, synergism, isobologram, *in vivo* studies

## Abstract

**BACKGROUND:**

The current chemotherapy for Chagas disease is based on monopharmacology with low efficacy and drug tolerance. Polypharmacology is one of the strategies to overcome these limitations.

**OBJECTIVES:**

Study the anti-*Trypanosoma cruzi* activity of associations of benznidazole (Bnz) with three new synthetic *T. cruzi-*triosephosphate isomerase inhibitors, **2**, **3**, and **4**, in order to potentiate their actions.

**METHODS:**

The *in vitro* effect of the drug combinations were determined constructing the corresponding isobolograms. *In vivo* activities were assessed using an acute murine model of Chagas disease evaluating parasitaemias, mortalities and IgG anti-*T. cruzi* antibodies.

**FINDINGS:**

The effect of Bnz combined with each of these compounds, on the growth of epimastigotes, indicated an additive action or a synergic action, when combining it with **2** or **3**, respectively, and an antagonic action when combining it with **4**. *In vivo* studies, for the two chosen combinations, **2** or **3** plus one fifth equivalent of Bnz, showed that Bnz can also potentiate the *in vivo* therapeutic effects. For both combinations a decrease in the number of trypomastigote and lower levels of anti-*T. cruzi* IgG-antibodies were detected, as well clear protection against death.

**MAIN CONCLUSIONS:**

These results suggest the studied combinations could be used in the treatment of Chagas disease.

Chagas disease, caused by the protozoan *Trypanosoma cruzi* (*T. cruzi*), represents a health threat for about 10-20 million people, being the second highest burden of disease among tropical diseases in the Americas (Nouvellet et al. 2015). The current chemotherapy is based on monopharmacology using nifurtimox (Nfx) or benznidazole (Bnz). They have limited efficacy and severe side effects (Castro et al. 2006). Some strategies to overcome the treatment limitations have included the development of new drugs, polypharmacologies and drug repositioning (Bahia et al. 2014). In the first approach, to identify new drugs, hundreds of compounds, from synthetic and natural sources, have been tested against *T. cruzi* (Cerecetto & González 2010, González & Cerecetto 2011). We recently described new compounds, belonging to different chemotypes, which were able to act *in vivo* decreasing the animal parasitaemia, i.e. compounds **1-4** (Fig. 1), surpassing the “hit-to-lead” drug discovery stage. They were designed as *T. cruzi* triosephosphate isomerase (Tc-TIM) inhibitors (Álvarez et al. 2015a, b, Aguilera et al. 2016) finding in some cases, i.e. derivatives **3** and **4**, the best results against this biological target. Although they displayed excellent *in vivo* behaviour some limitations were observed. For example, derivative **2** (Álvarez et al. 2015b), unlike derivative **1** at similar doses and administration regime (Álvarez et al. 2015a), showed limited survival rate of animals. On the other hand, derivatives **3** and **4**, unlike derivatives **1** and **2**, produced an increment of parasitaemia after the end of the treatment and limited survival rate of animals (Aguilera et al. 2016).

**Fig. 1:**
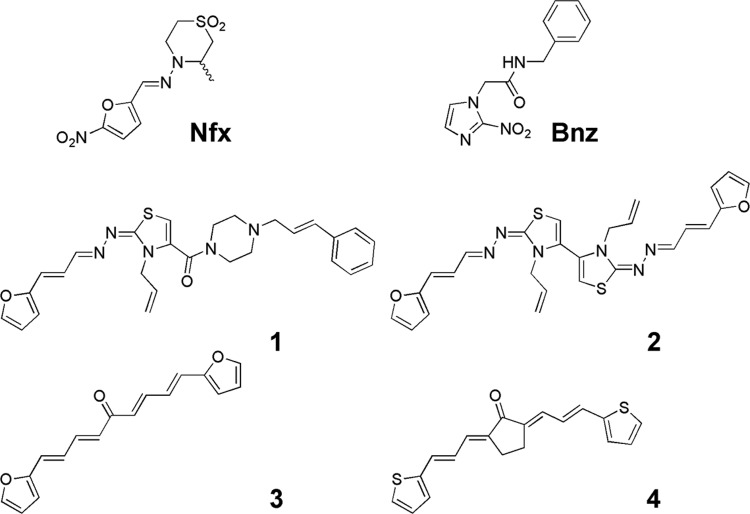
nifurtimox (Nfx), benznidazole (Bnz) and the *Trypanosoma cruzi* triosephosphate isomerase (*Tc*TIM) inhibitors with *in vivo* anti-*T. cruzi* activity described previously by our group (Álvarez et al. 2015a, b, Aguilera et al. 2016).

Concerning Chagas disease, evidences have grown in favour of the use of drugs combinations to enhance treatment efficacy and tolerance. These studies focused in the combination of different chemotypes with different parasitic point of actions trying to produce complete cure, reduce drug doses or diminish duration of the treatments. Some relevant examples are the drug repositioning approach using: anti-fungal agents combined with benznidazole (Araújo et al. 2000, da Silva et al. 2012, Diniz et al. 2013, Martins et al. 2015), combination of different anti-fungals (Urbina et al. 1988), anti-fungals combined with the inhibitor of 3-hydroxy-3-methylglutaryl-coenzyme A reductase lovastatin (Urbina et al. 1993), an anti-fungal agent combined with the anti-arrhythmic amiodarone (Benaim et al. 2006), an anti-fungal agent combined with an anti-tuberculosis drug (Veiga-Santos et al. 2015), suramin combined with Bnz (Santos et al. 2015), anti-inflammatory agents aspirin or simvastatin combined with Bnz or Nfx (López-Muñoz et al. 2010, Campos-Estrada et al. 2015), the glutathionylcysteine inhibitor L-buthionine (*S*,*R*)-sulfoximine combined with Nfx (Faúndez et al. 2008), and other combinations (Cencig et al. 2012). Although to a lesser extent, the use of new drugs, from synthetic or natural sources, combined with Bnz was also studied in the polypharmacology approaches (Pelizzaro-Rocha et al. 2010, Valdez et al. 2012, Rodrigues et al. 2014).

As part of our ongoing program (Cerecetto & González 2008) in the search of new molecules which could provide new lead compounds for Chagas disease treatment we found excellent *in vivo* prototypes, such as 2, 3, and 4 (Fig. 1), that require more study from a pharmacological point of view. In this sense, herein we describe the study of these compounds combined with Bnz as potential candidates for the treatment of Chagas disease.

## MATERIALS AND METHODS

*Compounds* - All chemicals were from Sigma (USA) or Merck (Germany). Compounds **2**, **3**, and **4** were synthesised as previously (Álvarez et al. 2015a, b, Aguilera et al. 2016). Bnz was purchased from LAFEPE (Pernambuco, Brazil).

*In vitro drug combination assay* - To verify the effect of the combination of thiadiazole **2** and Bnz or **3** and Bnz on epimastigotes we applied method previously described (Hallander et al. 1982, Urbina et al. 1988, 1993, Veiga-Santos et al. 2012). *T. cruzi* epimastigotes (Tulahuen 2 strain, discrete typing unit (DTU) Tc VI) were grown at 28°C in BHI-tryptose milieu supplemented with 5% foetal bovine serum. Cells from a 5-7-day-old culture were inoculated in fresh culture milieu to give an initial concentration of 1.00 × 10^6^ cells/mL. Cell growth was followed by measuring the absorbance of the culture at 600 nm every day. At day 5, the milieu was mixed with different concentrations of each compound combination, i.e. **2** and Bnz, **3** and Bnz or **4** and Bnz, dissolved in DMSO. The final concentration of DMSO in the culture milieu never exceeded 0.4%. No effect on epimastigotes growth was observed due to the presence of up to 1% DMSO in the culture milieu. Cultures containing non-treated epimastigote forms and 0.4% DMSO were included as negative controls. The different used concentrations of each compound combination were: 0.5 times IC_50,Bnz_ + IC_50,compound_; 0.5 times IC_50,Bnz_ + 0.75 times IC_50,compound_; 0.5 times IC_50,Bnz_ + 0.5 times IC_50,compound_; 0.5 times IC_50,Bnz_ + 0.25 times IC_50,compound_; IC_50,Bnz_ + 0.5 times IC_50,compound_; 0.75 times IC_50,Bnz_ + 0.5 times IC_50,compound_; 0.25 times IC_50,Bnz_ + 0.5 times IC_50,compound_; being IC_50,compound_ previously determined (Álvarez et al. 2015b, Aguilera et al. 2016). These mixtures were inoculated with 0.6 mL of a culture diluted to give a final parasite concentration of approximately of 1 × 10^6^ parasites/mL. After five days the percentage of growth inhibition (PGI) was calculated for each mixtures as follows: PGI (%) = {1 -[(Ap - A_0_p)/(Ac - A_0_c)]} × 100, where Ap = A_600 nm_ of the culture containing the drug at day 5, A_0_p = A_600 nm_ of the culture containing the drug just after addition of the inocula (day 0), Ac = A_600 nm_ of the culture in the absence of drug (control) at day 5, A_0_c = A_600 nm_ in the absence of the drug at day 0. Then the combination values (CVs) were graphically determined and each fractional inhibitory concentration (FIC) was calculated, according to Hallander et al. (1982), as the combined IC_50_ divided by the single IC_50_. The CV was defined as the concentrations of the compound combination permitting 50% of inhibition (PGI = 50%). The interaction index was calculated as follows: FIC = (IC_50_ compound in combination/IC_50_ compound alone) + (IC_50_ Bnz in combination/ IC_50_ Bnz alone). A FIC values less than, equal to, and more than 1 indicate synergism, additivity, and antagonism, respectively. The data were also graphically expressed as isobolograms, plotting the concentrations of each compound that combined produced a PGI = 50%. Each fractional dose was tested in triplicate and each antiproliferative experiment was done in duplicate.

*In vivo anti-T. cruzi activity (acute model)* - BALB/c male mice (30 days old, 25-30 g) bred under specific pathogen-free conditions, were infected by intraperitoneal injections of 5 × 10^3^ - 1 × 10^4^ blood trypomastigotes of the clone CL Brener (DTU Tc VI) (Álvarez et al. 2014, Álvarez et al. 2015a). First parasitaemias were counted six-eight days post-infection (week 1) and the treatment began five, six, or seven days post-infection (dpi), according our previous results when 80% of the animals were infected. In each experiment the mice were divided in two group (n = 6 or 7). One group of animals was used as control, that were inoculated orally with the vehicle (a lipid-based drug delivery system; Formariz et al. 2010, Álvarez et al. 2014), and the other group of animals was treated with the studied system: (1) compound **2** alone at 96.9 μmol (50 mg)/kg b.w./day; (2) combination of **2** at 96.9 μmol (50 mg)/kg b.w./day and Bnz at 19.2 μmol (5 mg)/kg b.w./day; (3) combination of **3** at 192.0 μmol (50 mg)/kg b.w./day and Bnz at 38.5 μmol (10 mg)/kg b.w./day; (4) Bnz alone at 19.2 μmol (5 mg)/kg b.w./day; (5) Bnz alone at 38.5 μmol (10 mg)/kg b.w./day; (6) Bnz alone at 96.2 μmol (25 mg)/kg b.w./day; (7) Bnz alone at 192.5 μmol (50 mg)/kg b.w./day; (8) Bnz alone at 385.0 μmol (100 mg)/kg b.w./day. Compounds were administered orally, via intragastric cannula, during 15 days in two different schemes. In the case of compound **2** and Bnz alone, and the combination of **2** with Bnz the dosages follow the schedule: three cycles of five days of administration with intervals of two days (treatment on 7-11 dpi, 14-18 dpi and 21-25 dpi for **2** alone and **2**+Bnz; treatment on 5-9 dpi, 12-16 dpi and 19-23 dpi for Bnz alone). In the case of combination of **3** and Bnz was administered for 15 consecutive days without breaks (treatment on 6-20 dpi). Parasitaemias in control and treated mice were determined once a week after the first administration, for 60 days post-beginning of treatment, in tail-vein blood. The number of parasites (trypomastigote forms) in blood was counted manually in an optical microscope (at 40× magnification). Additionally, the mortality rate was recorded. The anti-*T. cruzi* IgG-antibodies detections were done as: all the sera were obtained after centrifugation of the blood which was extracted from infected mice at 32 or 34 and 60 or 62 or 63 or 69 dpi. They were tested twice with an in-house ELISA kit (Chagas test, IICS, Asunción, Paraguay) following the procedure recommended by the manufacturer (IICS Production Department, Asunción-Paraguay). The optical density values were obtained in an ELISA plate reader (Titerek Unistan I). Student's *t*-test was used in order to compare both the parasitaemia and the levels of anti-*T. cruzi* antibodies between experimental groups.

The experimental protocols with animals were evaluated and supervised by the local Ethics Committee and the research adhered to the Principles of Laboratory Animal Care (Morton & Griffiths 1985).

## RESULTS

*In vitro assay of trypanosomicidal activities of drug associations against epimastigotes of T. cruzi* - In order to know the biological behaviour of each of the combinations, 2+Bnz, 3+Bnz, and 4+Bnz, we performed the corresponding *in vitro* isobolographic analysis.

**Fig. 2:**
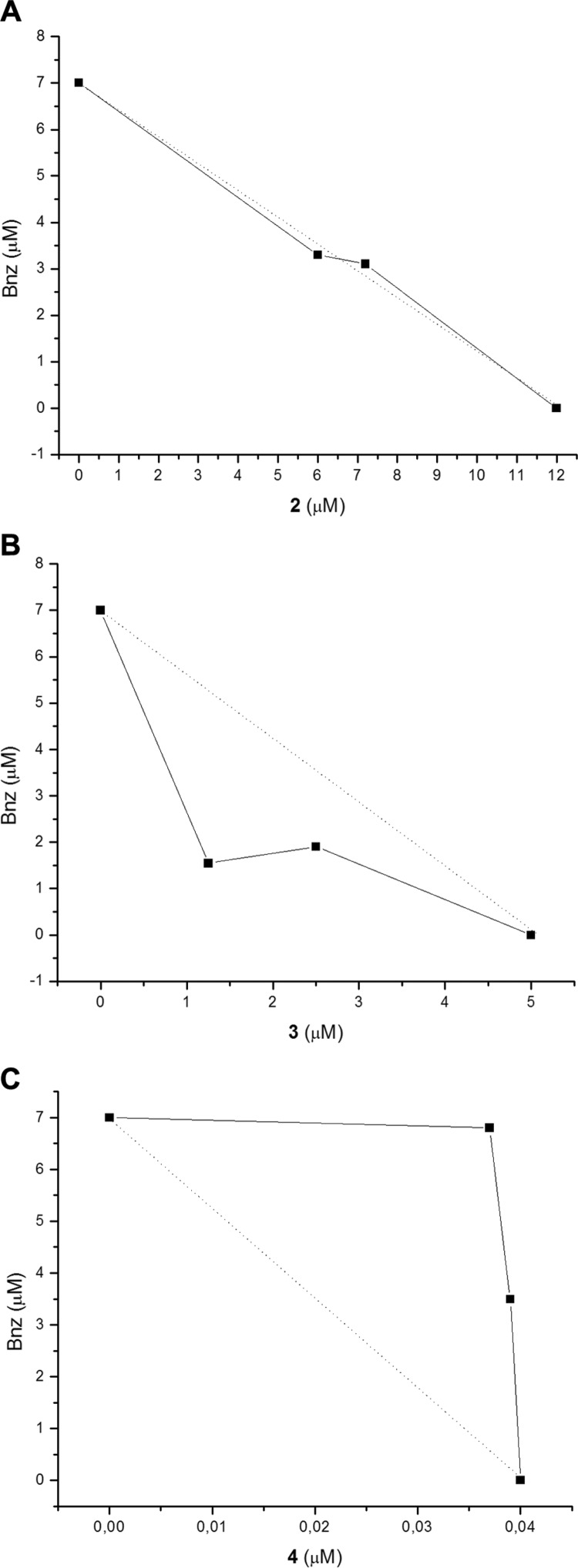
isobolographic analysis of the effect of benznidazole (Bnz) **2** (A), **3** (B) and **4** (C) and their combinations on epimastigotes. Dotted lines correspond to the predicted positions of the experimental points for additive effects and continuous lines correspond to the experimental findings. Points below the dotted line indicate a synergistic effect; points above the dotted line indicate an antagonistic effect. The experiments were repeated as it is indicated in Material and Methods section. The points show median values.

Compound **2** and Bnz, independently, have concentration-dependent effects on epimastigotes of Tulahuen 2 strain, DTU Tc VI, with an IC_50_ of 12.0 and 7.0 μM, respectively. The combination of **2** and Bnz did not result in synergistic or antagonistic effects and therefore have been classified as additive, with a fractional inhibitory concentration index (FICI) (Hallander et al. 1982) of 1.0. The graphical representation of this interaction is shown in the isobologram of Fig. 2A. Compound 3 showed also a concentration-dependent effect on epimastigote forms, with an IC_50_ of 5.0 μM (Aguilera et al. 2016). The combination of 3 and Bnz demonstrated promising results. In particular, a strong synergism against the epimastigotes was observed. As shown in Fig. 2B, the combination was below the additivity line (dotted line) on the isobologram with a FICI of 0.5. Also derivative **4**, displayed a concentration-dependent effect on the parasite with an IC_50_ of 40.0 nM (Aguilera et al. 2016). The combination of **4** and Bnz demonstrated an inadequate behaviour. The combination was above the additivity line on the isobolograph (Fig. 2C, dotted line) with a FICI of 2.0, thus indicating an antagonist effect.

These very interesting results revealed on one hand that the thiazole **2** could be combined with Bnz having an additive effect. On the other hand, addition of a low concentration of Bnz (one fourth of the IC_50_) reduces the IC_50_ of the furan **3** from 5.0 to 1.25 μM (reduction by 75%). Unfortunately, one of the products with the better activity when administered alone, furan **4**, was less active in combination with Bnz.

**Fig. 3:**
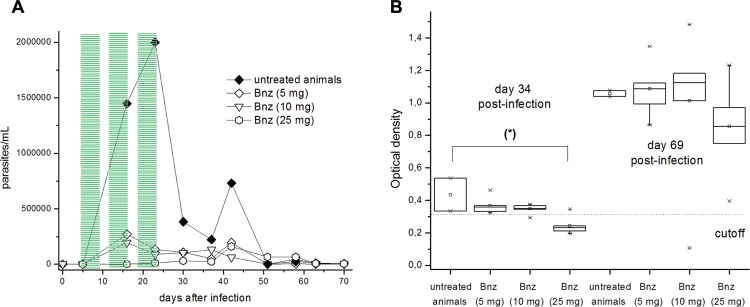
*in vivo* dose-response for benznidazole (Bnz). Course of infection of BALB/c male mice inoculated (i.p.) with 5 × 10^3^-1 × 10^4^ blood trypomastigotes of *Trypanosoma cruzi* clone CL Brener and orally treated with three cycles of five days with intervals of two days (5-9, 12-16, and 19-23 dpi). Experimental groups: 19.2 μmol (5 mg)/kg b.w./day of Bnz (◊), 38.5 μmol (10 mg)/kg b.w./day of Bnz (∇), 96.2 μmol (25 mg)/kg b.w./day of Bnz (⬡) and control group (infected and non-treated) (♦) (n = 6/group). (A) Parasitaemia curve. (B) Anti-*T. cruzi* IgG-antibodies levels in the different treatments. Cutoffs (dotted lines) correspond to anti-bodies levels for healthy animals (n = 3). Statistical significant differences in relation to the control group, evaluated by Student's *t*-test: (*) p < 0.03.

*In vivo proof of concepts* - In order to prove the pharmacological efficacy of the best combinations found in the *in vitro* assays, we evaluated *in vivo*, in a murine model of acute Chagas disease, the combination of **2** and Bnz and the combination of **3** and Bnz. For that, male BALB/c infected mice, with CL Brener clone, DTU Tc VI, were orally administered during 15 days with the combinations. In both cases the same dose of drug combination ratios was used, corresponding to a ratio (**2** or **3** *vs* Bnz) of 5 (one fifth, in μmol/kg, of Bnz). For compounds **2** and **3** we considered doses with intermediate *in vivo* activity (Álvarez et al. 2014, Aguilera et al. 2016). For Bnz we performed an *in vivo* study of dose-response varying the drug concentration between 19.2 to 385.0 μmol (10 to 100 mg)/kg b.w./day (Fig. 3). From this, we selected, for the further drug combinations, those doses where Bnz had no statistically significant effects on the levels of anti-*T. cruzi* antibodies (Fig. 3B).

The selected combinations were dosed in a micro-emulsion as vehicle which previously demonstrated good bioavailability with these kind of compounds (Álvarez et al. 2014, Aguilera et al. 2016). The courses of infections were monitored by counting blood parasites, animal mortality, and the anti-*T. cruzi* IgG-antibodies levels (in the middle and end of the assay).

**Fig. 4:**
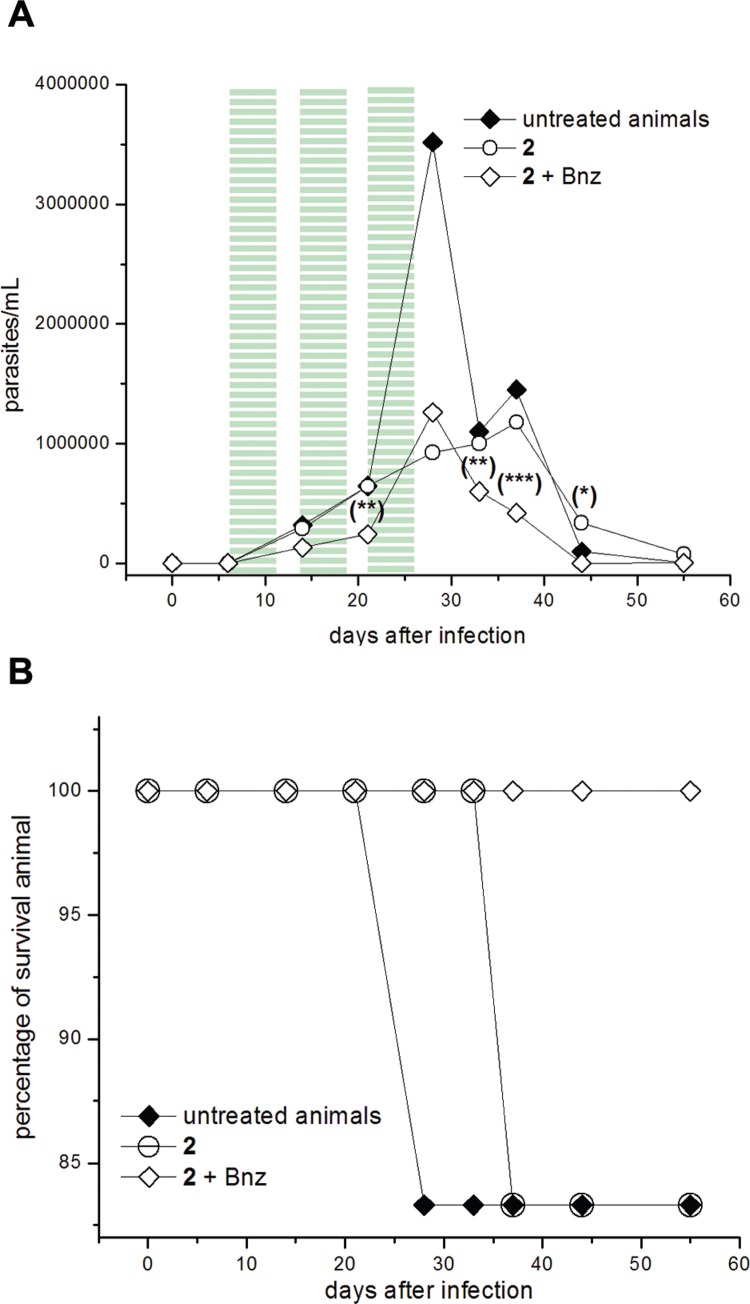
*in vivo* proof of concept. Course of infection of BALB/c male mice inoculated (i.p.) with 5 × 10^3^-1 × 10^4^ blood trypomastigotes of *Trypanosoma cruzi* clone CL Brener and orally treated with three cycles of five days with intervals of two days (7-11, 14-18, and 21-25 dpi). Experimental groups: 96.9 μmol (50 mg)/kg b.w./day of thiazole **2** (○), 96.9 μmol (50 mg)/kg b.w./day of thiazole **2** plus 19.2 μmol (5 mg)/kg b.w./day of Bnz (◊) and control group (infected and non-treated) (♦) (n = 6/group). (A) Parasitaemia curve. Highlighted regions (≡) correspond to the periods of treatment. Statistical significant differences in relation to the control group, evaluated by Student's *t*-test: (***) p < 0.03, (**) p < 0.05, (*) p < 0.06. (B) Survival curve.

**Fig. 5:**
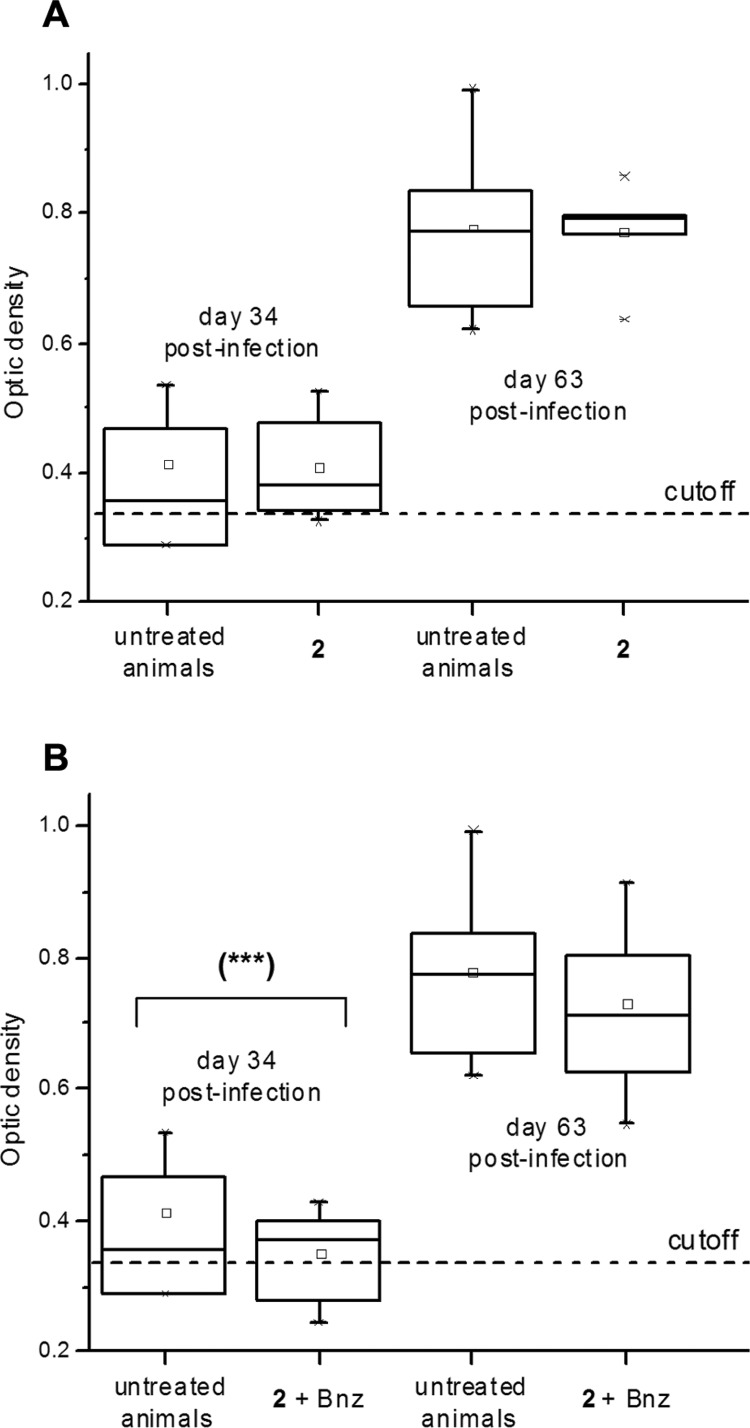
anti-*Trypanosoma cruzi* IgG-antibodies levels in the different treatments. Compound **2** alone (A) and the combination of **2** plus benznidazole (Bnz) (B), with the corresponding controls of untreated animals. Cutoffs (dotted lines) correspond to anti-bodies levels for healthy animals (n = 3). Statistical significant differences in relation to the control group, evaluated by Student's *t*-test: (***) p < 0.05.

Thiazole **2**, at 96.9 μmol (50 mg)/kg b.w./day, was administered together with one fifth equivalent of Bnz, 19.2 μmol (5 mg)/kg b.w./day, during five days of administration followed by two days of rest and repeating this administration-scheme two more times (Fig. 4A). We selected a lower dose of Bnz in order to produce the desired biological results without side effects. The addition of this little amount of Bnz in the dosage improved the profile of parasitaemia decreasing significantly the parasite load (days 21, 33, 36, and 44, Fig. 4A), compared to the treatment with compound **2** alone. The combination **2**+ Bnz was able to abolish the second maximum peak of parasitaemia (day 37). The survival rate for the thiazole **2** alone was, at the end of the assay, the same of the untreated animals (Fig. 4B) with a shift of the first day of death (from day 29, for the untreated animals, to day 35 for those treated with compound **2**). When the combination of **2** plus Bnz was dosed the mice survival was improved to 100% at the end of the assay. The combination of **2** plus Bnz was able to decrease significantly the anti-*T. cruzi* antibodies in the first quantification compared to untreated animals (day 34, Fig. 5B). Additionally, in this measuring point, unlike treatment with **2** alone (Fig. 5A) or untreated control, antibodies levels for some animals treated with the combination **2** + Bnz dropped below the cutoff (antibodies levels for healthy animals).

**Fig. 6:**
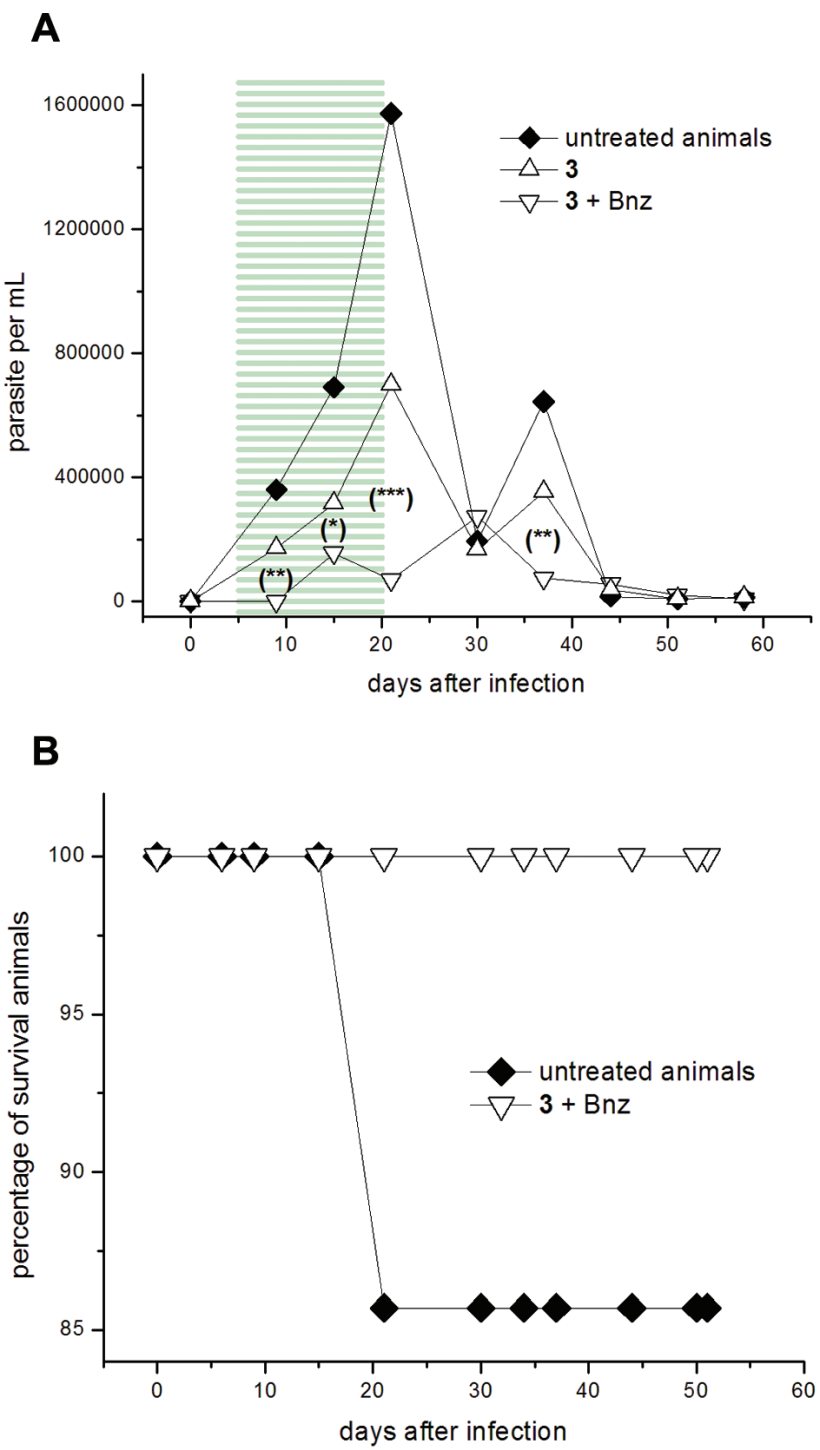
*in vivo* proof of concept. Course of infection of BALB/c male mice inoculated (i.p.) with 5 × 10^3^-1 × 10^4^ blood trypomastigotes of *Trypanosoma cruzi* clone CL Brener and orally treated for 15 consecutive days from 6 to 20 dpi. Experimental groups: 192.0 μmol (50 mg)/kg b.w./day of furan **3** (Δ) (from reference Aguilera et al. 2016), or 192.0 μmol (50 mg)/kg b.w./day of the furan 3 plus 38.5 μmol (10 mg)/kg b.w./day of Bnz (◇) and control group (infected and non-treated) (♦) (n = 7/group). (A) Parasitaemia curve. Highlighted regions (≡) correspond to the period of treatment. Statistical significant differences in relation to the control group, evaluated by the Student's *t*-test: (***) p < 0.03, (**) p < 0.05, (*) p < 0.06). (B) Survival curve.

**Fig. 7:**
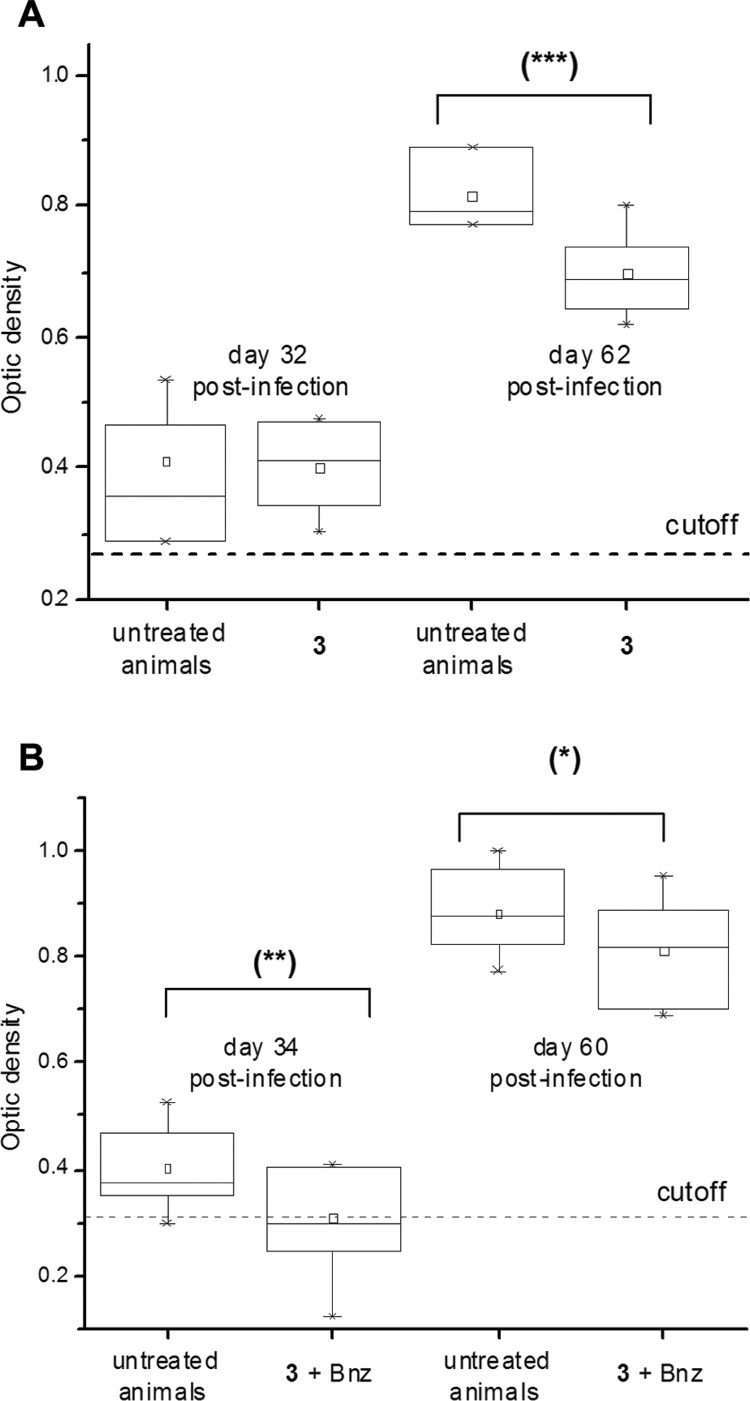
anti-*Trypanosoma cruzi* IgG-antibodies levels in the different treatments. Compound **3** alone (A) and the combination of **3** plus benznidazole (Bnz) (B), with the corresponding controls of untreated animals. Cutoffs (dotted lines) correspond to anti-bodies levels for healthy animals (n = 3). Statistical significant differences in relation to the control group, evaluated by Student's *t*-test: (***) p < 0.06, (**) p < 0.10, (*) p < 0.20.

Furan **3**, at 192.0 μmol (50 mg)/kg b.w./day, was administered together with one fifth equivalent of Bnz, 38.5 μmol (10 mg)/kg b.w./day, during 15 days of administration (Fig. 6A). The addition of this little amount of Bnz in the dosage improved the profile of parasitaemia decreasing significantly the parasite load in the combined treatment (days 9, 15, 21, and 37, Fig. 6A) when compared to the treatment with compound **3** alone. The combination **3** + Bnz was able to shift the first maximum parasitaemia peaks, since days 21 to day 30, abolishing the second one (day 37). Furthermore, the survival rate for the combination **3** + Bnz was better than the untreated animals (Fig. 6B) and those for the treatment with **3** alone (83% of animal survival at the end of the assay) (Aguilera et al. 2016). On the other hand, the combination of **3** + Bnz was able to diminish significantly the anti-*T. cruzi* anti-bodies levels compared to untreated animals, since the first check point (day 34, Fig. 7B). Moreover, in this first titration (day 34) unlike treatment with **3** alone (Fig. 7A) or untreated control, antibodies levels for a lot of animals in the treatment with the combination **3** + Bnz dropped below the cutoff (57% of the treated animals).

## DISCUSSION

The performed studies contribute to the use of the combination of Bnz with new chemical entities, in Chagas disease treatment. The three selected chemical entities, thiazole **2**, furan **3**, and thiophene **4**, previously developed and biologically studied by our group (Álvarez et al. 2015b, Aguilera et al. 2016), were chosen because they showed some slight disadvantage in the *in vivo* studies. It was intended to deal with these problems using the procedure of drug combinations.

For that, firstly all compounds **2**, **3**, and **4** were studied *in vitro* combined with Bnz. When using the combination of the thiazole **2** and Bnz an additive effect against the epimastigotes growth was observed while a synergic action was observed when we combined the furan **3** and Bnz, and an antagonic action, when we combined the thiophene **4** and Bnz.

Secondly, we performed the *in vivo* proof of concepts using an acute murine model of Chagas' disease analysing the two *in vitro* adequate combinations, **2** plus Bnz and **3** plus Bnz. The pharmacologic schedules involved the combination of one equivalent of **2**, or **3**, plus one fifth equivalent of Bnz. These combinations improved considerably the biological behaviour of the compounds **2** or **3** compared to when they were administered alone.

On the one hand, it was clearly evidenced that the trypomastigote loads for treatments, **2** + Bnz or **3** + Bnz, and during the experiments were lower than those for treatments with the compounds alone. The parasitic loads evolution showed that the synergic combination **3** + Bnz had the best profile with abolishment of both maximum parasitaemia peaks, at days 21 and 37, present in untreated animals.

On the other hand, both combined treatments, **2** + Bnz and **3** + Bnz, produced complete protection against death showing the ability of Bnz to potentiate the action of **2** and **3**. Similarly, the anti-*T. cruzi* IgG-antibodies levels, at 34 days after infection, were significantly decreased when Bnz was co-administered with thiazole **2** or to furan **3**.

Further pharmacological studies, modifying schedules of dosages, are currently in progress. The promising results obtained, as additive or synergistic interactions of compounds with different mode of actions, suggest that they could be developed as pharmacologic strategies for Chagas disease treatment.
